# Recurrence of Epidural Spinal Sarcoidosis

**DOI:** 10.5435/JAAOSGlobal-D-21-00086

**Published:** 2021-07-16

**Authors:** Louis A. Magdon, Robin Elliott, Christina W. Cheng

**Affiliations:** From the Department of Orthopaedic Surgery (Dr. Magdon and Dr. Cheng), University Hospitals Cleveland Medical Center, Cleveland, OH, and the Department of Pathology (Dr. Elliott), University Hospitals Cleveland Medical Center, Cleveland, OH.

## Abstract

Neurosarcoidosis involving the spine is uncommon. Sarcoidosis of the spine usually presents as an intramedullary lesion and rarely an epidural lesion. To have recurrence of neurosarcoidosis is an even rarer presentation. Here, we present a 37-year-old man with poorly controlled sarcoidosis who initially presented to our medical center in 2015 with thoracic myelopathy from epidural spinal sarcoidosis treated with thoracic decompression and fusion. He presented to the hospital 5 years later with a month history of progressive upper extremity weakness. MRI revealed recurrent stenosis and spinal cord compression in the cervicothoracic junction. Urgent surgical intervention along with medical management resulted in symptomatic and functional improvement. Surgical intervention and compliance with postoperative corticosteroid therapy seem to yield a favorable prognosis for patients with epidural spinal sarcoidosis and to avoid recurrence.

Sarcoidosis is a systemic disease of unknown etiology characterized by granulomatous inflammation. It most commonly affects the lungs, skin, and eyes but can also manifest in other organs.^1^ Sarcoidosis of the spine is a rare presentation. Several case reports have documented the presentation of sarcoidosis in the vertebral bodies.^2,3,4,5,6,7,8,9,10,11,12^ However, cases of intraspinal sarcoidosis have been reported but are almost exclusively that of intradural spinal cord disease.^13,14,15,16,17,18,19^ There are eight reported cases of intraspinal epidural sarcoidosis published.^20,21,22,23,24,25,26,27^ Most cases present in the thoracic spine^20,21,23,24,25,26^ with one reported case in the lumbar spine^27^ and one in the cervical spine.^24^ To the best of our knowledge, the reporting of recurrent intraspinal epidural sarcoidosis in the spine has only been reported once in a pediatric patient. Thus, we report a unique case of intraspinal epidural sarcoidosis in an adult patient presenting with recurring sarcoidosis-associated myelopathy in both the thoracic spine and the cervical spine on two separate presentations 5 years apart.

## Case Report

A 37-year-old African American man presented to our emergency department with 1-month history of progressive bilateral upper extremity weakness, with his right side worse than his left and gait instability. He did not report urinary or bowel incontinence. He has a medical history of poorly controlled sarcoidosis, with a known history of uveitis, pulmonary nodules, mediastinal adenopathy, and lytic hand lesions. He had stopped his medications approximately 1 year before his presentation.

**Figure 1:**
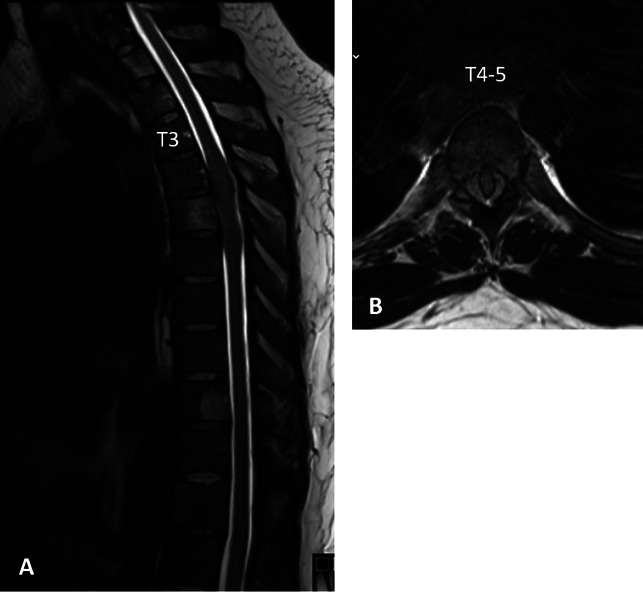
Radiographs showing sagittal T2 MRI (**A**) and axial cross section at the level of T4-5 (**B**) from the patient's initial presentation 5 years before recent presentation showing an epidural mass from T4-5.

Approximately 5 years before the current presentation, he presented to our emergency department with back pain and progressive bilateral lower extremity weakness, paresthesia, and gait disturbance. He had a normal motor examination except for 4/5 grade strength with left hip flexion with diffuse paresthesia over the entire left leg. Perianal sensation and rectal tone were intact with negative Hoffman sign, Babinski, and clonus. MRI revealed a thoracic epidural mass compressing the spinal cord at T4-T6 (Figure 1). A CT-guided biopsy of the left paraspinal intramuscular mass was done to rule out malignancy. Tissue diagnosis revealed nonnecrotizing granulomatous inflammation. He was treated surgically with instrumented posterior spinal fusion from T3 to T6, a T4 to T6 laminectomy and decompression, and epidural mass debulking. Sarcoidosis was confirmed as the final tissue diagnosis. Postoperatively, he was started on methylprednisolone and transitioned to prednisone before discharge. Per rheumatology recommendation, he was on prednisone 60 mg daily for 2 weeks and then 40 mg daily for 2 weeks and 30 mg daily continuously. At his 3-week postoperative visit, he had full strength in his lower extremity with a stable gait. He was lost to follow-up after his 3-month postoperative visit.

**Figure 2:**
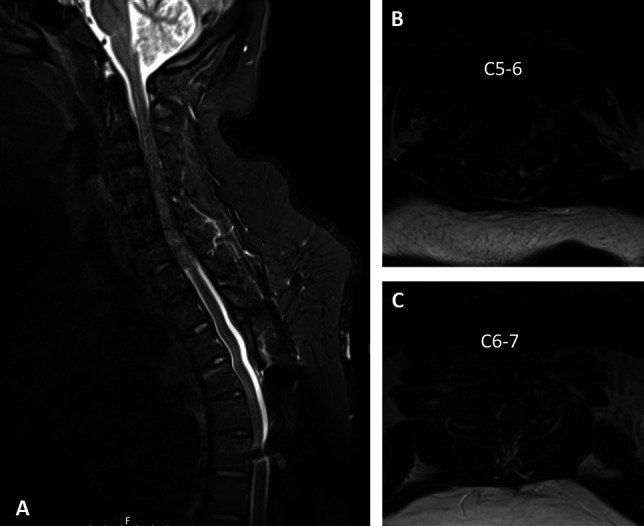
Radiographs showing sagittal short tau inversion recovery magnetic resonance imaging of the cervical and thoracic spine (**A**) and T2 axial cross sections at C5-6 (**B**) and C6-7 (**C**) demonstrating recurrent epidural mass from C4-T1.

At his present visit, he had an ataxic gait, manual muscle testing showed 5/5 bilaterally in the deltoid and biceps, 4/5 bilaterally with wrist extension, 4/5 in the triceps on the left and 3/5 in the triceps on the right, 4/5 bilaterally with finger flexion, and 3/5 in the interosseous muscles of hands bilaterally. Inspection of the upper extremities revealed intrinsic wasting of his hands and atrophy of his forearms, with right being greater than left. He also had pain and swelling of both hands over his interphalangeal joints. In addition, soft-tissue nodules were present on the right side of the posterior aspect of his neck and over his previous surgical spine incision. Upper motor neuron signs were not present in his bilateral upper and lower extremities. His perianal sensation was intact, and he had normal rectal tone. MRI of the entire spine with and without contrast showed enhancing soft tissue extending along the right lateral and posterior epidural space from C4 to T1, causing severe stenosis and mass effect and leftward deviation of the spinal cord. The epidural tissue extends into the right C4-5, C5-6, C6-7, and C7-T1 neural foramina. There are T2-hyperintense signal changes within the cord at the level of C5-6 and C6-7, suggestive of myelomalacia (Figure 2). There was no evidence of epidural tissue causing stenosis in the remainder of the spine.

He was initially treated with intravenous Decadron 10 mg every 8 hours for 24 hours, for which he reports improvement in hand paresthesia. Given the patient's symptoms of progressive upper extremity weakness with imaging findings of cervical stenosis and spinal cord compression from likely epidural sarcoidosis, he was recommended urgent surgical intervention which he agreed to after discussing the risks, benefits, and expectations. Before surgery, pulmonology, rheumatology, and medicine service were consulted for medical optimization because patient is at risk of cardiopulmonary sarcoidosis as a former smoker with a known history of pulmonary sarcoidosis. He underwent chest CT that showed mediastinal adenopathy and multiple small pulmonary nodules in bilateral upper lobes without parenchymal disease.

**Figure 3:**
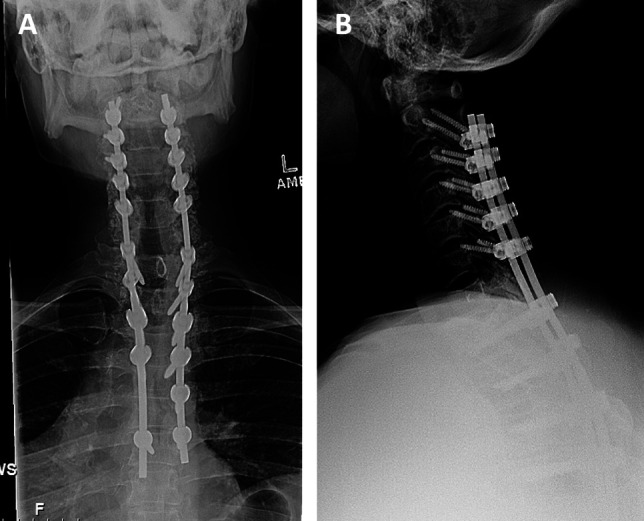
Postoperative standing upright plain radiograph showing cervicothoracic spine AP (**A**) and lateral (**B**) views.

**Figure 4:**
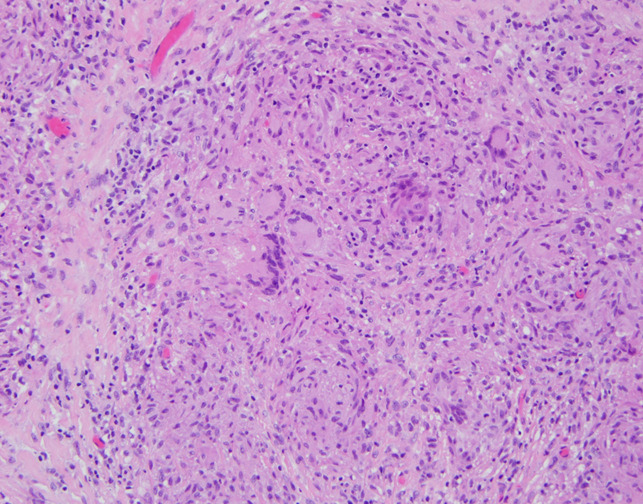
Histology slides from intraoperative tissue biopsy stained with hematoxylin and eosin showing nonnecrotizing granulomas containing multinucleated giant cells ×200.

The patient was surgically treated with C3-C7 laminectomy with extension of his previous fusion from C2 to T6. The posterior spine was exposed from C2 to the previous fusion at T3-T6. Exposure of the previous surgical site revealed a solid fusion mass from T3 to T6 bilaterally with adequate fixation of all previously placed pedicle screws. Instrumentation was placed from the previous fusion to C2 to adequately stabilize the cervicothoracic spine for the necessary decompression (Figure 3). Laminectomies were done using a bilateral trough technique from C3 to C7. After the laminectomy, visual inspection revealed a layer of epidural tissue surrounding and compressing the thecal sac. The epidural tissue was removed and sent for tissue diagnosis. A layered wound closure was done. An incisional wound negative pressure dressing was used to prevent potential wound infection or wound healing complications, given that we had to incise through his previous incision that healed with keloid-like scar tissue. He was placed in an Aspen cervical collar after the surgery. Postoperatively, the patient received Decadron 10 mg every 8 hours for 24 hours and transitioned to oral prednisone 40 mg daily as per rheumatology recommendations. Negative pressure dressing was maintained for a total of 2 weeks to help with wound healing. Pathology review of the epidural mass biopsy revealed nonnecrotizing granulomas consistent with sarcoidosis as the tissue diagnosis (Figure 4). At his 3-week postoperative visit, the patient regained full strength in his upper extremities and intrinsic, paresthesia in his hands resolved, and he had full sensation. The patient also demonstrated improved ambulation and ability to walk heel-and-toe without instability. His incision healed without complications.

## Discussion

Neurosarcoidosis of the spine is a rare presentation of this systemic disease characterized by granulomatous inflammation.^1^ A large number of case reports on neurosarcoidosis are available in the literature. Approximately 5% of all sarcoidosis involve the central nervous system, with most being intracranial.^22,26^ Only 10% of neurosarcoidosis involve the spine.^22,26^ Sarcoidosis has been described to manifest both in the vertebral bodies and within the spinal canal.^2,3,4,5,6,7,8,9,10,11,12,13,14,15,16,17,18,19,20,21,22,23,24,25,26^ Those with intraspinal presentation are almost exclusively intradural sarcoidosis.^13,14,15,16,17,18,19,20,21^ To the best of our knowledge, there are nine reported cases of intraspinal epidural sarcoidosis in the literature, including ours (Table 1).

**Table 1:** Reported Cases of Intraspinal Epidural Neurosarcoidosis

Authors	Age/Sex	Symptoms	Localization	Systemic Involvement	Surgical Management	Medical Management	Response to Therapy
Weissman et al., 1996^27^	37M	LE radicular pain, paresthesias, and difficulty voiding	L1-S1	Paratracheal adenopathy	L1-S1 bilateral laminectomy and excision of epidural mass.	Postoperative prednisone for 4 mo and gradually weaned off.	Immediate relief within days of surgery. Asymptomatic at 7 mo postoperatively.
Nardon et al., 2006^25^	80F	Right hemiplegia and left sided hyperesthesia	C2-C3	Lung with hylar and mediastinal lymphadenopathy, anterior uveitis	None	250 mg/day methylprednisolone for 5 days, followed by 40 mg daily prednisone, and gradually tapered over 2 mo.	Improve neurologic function and resolution of epidural mass on MRI at 2 mo postoperatively.
Nardon et al., 2006^25^	52F	Back pain, numbness, weakness in LE, and difficulty with micturition	T2-T6	None	Thoracic laminectomy and biopsy of epidural mass	Postoperative 1g/day for 5 days of methylprednisolone, followed by daily 60 mg prednisone for 7 days and reduced to 20 mg daily.	Complete neurologic recovery. Resolution of epidural mass on MRI at 1 year.
Barazi et al., 2008^20^	44F	Back pain and LE paresthesia	T12-L1	None	Bilateral T1-L1 laminectomies and excision of lesion.	Not described	Neurologically intact without radiographic recurrence at 4 mo.
Galgano et al., 2018^21^	9F	LE weakness, bilateral thigh pain and episodic urinary incontinence with urinary retention. Sensory deficit below T6.	1. T3-T6 2. Recurrence at 3 mo from T3-T7 3.Recurrence at 5 mo at T1-T8	FDG-PET scan with uptake in axial and appendicular skeleton	1. Left T3-T4 hemilaminotomy and debulking of epidural mass 2. T5-T6 laminectomy and debulking at 3 mo after 1^st^ surgery.	1. Postoperative steroids 2. Postoperative steroids and methotrexate 3. Restarted on methotrexate and steroid after second recurrence.	1. Resolution of symptoms 1 week after start of steroids after first episode. 2. 1 year after initial presentation with normal neurologic examination.
Munakomi et al., 2018^24^	50M	LE weakness and sensory loss below T7.	T3-T4 T9-T10	Multiple fibrotic changes in right lung	Thoracic laminectomy and subtotal resection of the epidural mass.	Prednisolone taper and weekly methotrexate therapy	Ambulating with cane within 10 days and independently at 4 weeks.
Paglia et al., 2019^26^	45F	Back pain and LE paresthesia and weakness.	T11-L1	Right lung nodule, lymphadenopathy of the mediastinal lymph nodes, and uterine fibromatosis.	T11-L1 bilateral laminectomy and excision of extradural lesion.	Methylprednisolone 1 mg/kg daily for 3 days, followed by prednisone 1 mg/kg daily.	Progressive improvement in symptoms. CT scan with complete disease remission 6 mo postoperative.
Longo et al., 2019^23^	46F	Low back pain, unsteady gait, and sensory loss below knees.	T4-T6, T8 L2-L3	None	None	Bolus of 10 mg dexamethasone, followed by 4 mg every 6 hours. Discharged on prednisone taper for 4 mo.	Improve back pain and gait at 1 week and remained neurologically intact and ambulating without difficulty at 18 mo.
Khairy et al., 2020^22^	29F	Dysphagia, neck pain, and cervical myeloradiculopathy	C2-C7	None	None	Preoperative dexamethasone 4 mg every 6 hours. Discharged with daily prednisolone taper more than 8 weeks	Clinical improvement at 3 mo.
Our case	37M	Bilateral UE weakness and unsteady gait.	1. T4-T6 2. C4-T1	Mediastinal adenopathy with multiple pulmonary nodules in bilateral lungs. Hand lesions and uveitis.	1. T3-T6 laminectomy and fusion 2. C3-C7 laminectomy with C2-T6 fusion.	1. Postoperative methylprednisolone and prednisone taper. 2. Preoperative 10 mg Decadron every 8 hours for 24 hrs. Postoperative Decadron 10 g every 8 hours for 24 hours. Discharge on prednisone taper over 3 mo.	Complete neurologic improvement at 3-week follow-up and continues to do well at 3 mo.

FDG-PET = Fludeoxyglucose Positron Emission Tomography, LE = lower extremity, UE = upper extremity

Our case is unique in that our patient developed recurrence of intraspinal epidural spinal sarcoidosis-associated myelopathy. Approximately 5 years before his recent presentation, he came to medical attention for epidural spinal sarcoidosis-associated thoracic myelopathy. He endorsed progressive weakness in his legs and was treated with a thoracic decompression and instrumented fusion in addition to steroids. His neurologic symptoms improved after surgery, but unfortunately, he did not comply with the recommended systemic treatment, and it was likely the cause of his recurrent epidural spinal sarcoidosis. MRI from 5 years ago did show anterior epidural soft tissue at the C5-6 level with marrow replacement involving the C6, thoracic, and lumbar vertebra; however, there was no evidence of cord compression at that time. With the absence of systemic treatment, this allowed the epidural tissue to expand and grow.

On presentation to us, our patient endorsed progressive upper extremity weakness for approximately 1 month. Given his medical history of poorly controlled sarcoidosis and surgical treatment of epidural spinal sarcoidosis-associated thoracic myelopathy, the presence of a compressive cervical epidural mass on imaging with MRI increased our suspicion for a diagnosis of recurrence of epidural spinal sarcoidosis. Given the presence of a progressive neurologic deficit and the successful outcome of the previous surgical intervention for his epidural disease, the patient was recommended surgical intervention again in addition to preoperative and postoperative corticosteroids as a treatment for which he consented.

As with our case, seven of the eight cases of intraspinal epidural sarcoidosis presented with neurologic deficits, and surgical treatments were undertaken in each with improvement in their neurologic symptoms.^20-22,24,25,26,27^ After surgical decompression, each patient underwent medical management with steroid taper^20-22,24,25,26,27^ and two patients had additional methotrexate therapy.^21,24^ Longo et al.^23^ reported a patient with multiple epidural lesions at T4-T6, T8, and L2-L3 who only received medical management, initially with 10 mg bolus of dexamethasone followed by 4 mg dexamethasone every 6 hours. She was then discharged with oral prednisone taper over a 4-month period. At 1 week after discharge, her gait had improved markedly, and at 18 months after initial admission, she was ambulating without difficulty, neurologically intact, and employed.^23^ Similarly, Nardon et al.^25^ also reported a case of an 80-year-old woman with cervical neurosarcoidosis who was also managed with 2 months of steroids with improved neurologic function and MRI demonstrating a resolution of epidural mass.

Galgano et al.^21^ reported one case of neurosarcoidosis in a pediatric patient who developed recurrent neurosarcoidosis due to noncompliance with medical treatment similar to our patient. They presented a case of a 9-year-old girl with rapid onset thoracic myelopathy who was experiencing progressive lower extremity weakness with falls, urinary symptoms, and bilateral thigh pain over the course of 2 weeks. MRI noted extensive circumferential enhancing epidural mass from T3-6 with severe spinal cord compression and cord signal change, and an emergent open biopsy was done using a left T3-4 hemilaminotomy and debulking of the epidural mass. Corticosteroids were started in the immediate postoperative period for a short postoperative course. Within a week, her neurologic deficit had improved. She would go on to have a couple of recurrences within months, the first recurrence treated with additional thoracic laminectomies and mass debulking and the second recurrence managed medically, with the first recurrence noted after only a short course of corticosteroids was given and the second recurrence noted from noncompliance with medical therapy. One year after the initial presentation, she was noted to have normal function.

In the eight cases of epidural spinal sarcoidosis, including ours, all of whom presented with a neurologic deficit, a combination of surgical intervention and medical therapy ultimately resulted in an improvement of their neurologic deficit and thus yielded a favorable outcome. In addition, our case and the case reported by Galgano et al. shed light on the importance of postoperative medical therapy because our patient had demonstrated that noncompliance with medical therapy resulted in the recurrence of neurosarcoidosis. However, the case reports of epidural spinal sarcoidosis are not consistent with those of intradural diseases regarding steroid treatment and recurrence. Some cases used postoperative steroid treatment, but others did not, and regardless of whether steroids were given, no recurrence was observed.^14,16,17^

In conclusion, epidural spinal sarcoidosis is rare. From the cases reported in the literature, including our patient, they all presented with a neurologic deficit that improved with a combination of surgical intervention and medical therapy, consisting mainly of corticosteroids. Two of the cases stress the importance of postoperative systemic therapy to prevent recurrence of the epidural disease. Surgical intervention and compliance with postoperative corticosteroid therapy seem to yield a favorable prognosis for patients with epidural spinal sarcoidosis.
